# An improved DNA array-based classification method for the identification of *Salmonella* serotypes shows high concordance between traditional and genotypic testing

**DOI:** 10.1371/journal.pone.0207550

**Published:** 2018-12-04

**Authors:** James Robertson, Catherine Yoshida, Simone Gurnik, Madison McGrogan, Kristin Davis, Gitanjali Arya, Stephanie A. Murphy, Anil Nichani, John H. E. Nash

**Affiliations:** 1 Public Health Agency of Canada, National Microbiology Laboratory, Guelph, Ontario, Canada; 2 Public Health Agency of Canada, National Microbiology Laboratory, Winnipeg, Ontario, Canada; Defense Threat Reduction Agency, UNITED STATES

## Abstract

Previously we developed and tested the *Salmonella* GenoSerotyping Array (SGSA), which utilized oligonucleotide probes for O- and H- antigen biomarkers to perform accurate molecular serotyping of 57 *Salmonella* serotypes. Here we describe the development and validation of the ISO 17025 accredited second version of the SGSA (SGSA v. 2) with reliable and unambiguous molecular serotyping results for 112 serotypes of *Salmonella* which were verified both *in silico* and *in vitro*. Improvements included an expansion of the probe sets along with a new classifier tool for prediction of individual antigens and overall serotype from the array probe intensity results. The array classifier and probe sequences were validated *in silico* to high concordance using 36,153 draft genomes of diverse *Salmonella* serotypes assembled from public repositories. We obtained correct and unambiguous serotype assignments for 31,924 (88.30%) of the tested samples and a further 3,916 (10.83%) had fully concordant antigen predictions but could not be assigned to a single serotype. The SGSA v. 2 can directly use bacterial colonies with a limit of detection of 860 CFU/mL or purified DNA template at a concentration of 1.0 x 10^−1^ ng/μl. The SGSA v. 2 was also validated in the wet laboratory and certified using panel of 406 samples representing 185 different serotypes with correct antigen and serotype determinations for 60.89% of the panel and 18.31% correctly identified but an ambiguous overall serotype determination.

## Introduction

*Salmonella* is a common foodborne zoonotic pathogen of public health concern and it is the leading cause of bacterial gastroenteritis in North America with an estimated 1.02 million cases of non-typhoidal *Salmonella* in the United States [1] and 87,500 in Canada annually [2]. There is a great deal of interest within the public health community to developing faster and higher resolution diagnostics for *Salmonella* due to the significant burden it represents to both the medical and economic resources world-wide. The *Salmonella* genus consists of two species *enterica* and *bongori* with five subspecies described within the *enterica* species: I) enterica, II) salamae, IIIa) arizonae, IIIb) diarizonae, IV) houtenae and V) indica [3]. The gold-standard methodology for classification of serotypes of *Salmonella* is phenotypic serotyping of isolates based on the reaction of antisera to surface antigens. Isolates are categorized into serotypes based on the combination of somatic (O) antigens of the lipopolysaccharide layer (LPS) and flagellar (H) antigens according to the White-Kauffman-Le Minor (WKL) scheme [3,4]

Phenotypic serotyping forms the basis of global surveillance of *Salmonella* and there are numerous implications to the presence of specific serotypes, including public health and food safety, outbreak management, and even trade [5,6]. Serotyping has served as a valuable tool in the characterization of *Salmonella* but it requires specialized labor, maintenance of antisera, and is not amenable to high-throughput [7,8]. Furthermore, the time required for full serotyping of samples can take 4–5 days which delays investigations [7,8]. There are 46 somatic serogroups within *Salmonella* and 114 flagellar antigens[4], each requiring one or more specific antisera for identification, thus representing a large number of antisera needed to be maintained. Numerous molecular replacements for serotyping have been developed to address some of the shortfalls of phenotypic serotyping [7–9].The *Salmonella* genoserotyping array (SGSA) was developed by the Public Health Agency of Canada (PHAC) to address some of the issues associated with traditional serotyping and has been found to be highly reliable [10].

Building on the successes of the SGSA v. 1 and upon our own experiences deploying it into the OIE (World Organisation for Animal Health) *Salmonella* Reference Laboratory, Guelph, Ontario, Canada, we have updated and improved the technology so that it can be more readily incorporated into laboratory workflows and increased the capacity of the array to identify more serotypes such as *Salmonella enterica* ser. Enteritidis that are commonly implicated in food safety.

## Materials and methods

### Probe development and assay layout

All newly developed probes and primers used in the development of the assay were designed using the PrimerSelect (DNASTAR, Madison, WI) software and specificity of the targets were confirmed using Basic Local Alignment Search Tool (blastn) from NCBI, as described (8]. Each probe was printed in triplicate onto Alere ArrayTube strips (Alere Technologies GmbH, Germany) which are now commercially available. The *Salmonella* specific gene *invA* was used as a positive control for the assay since it is rare for a *Salmonella* to be *invA* negative and the gene is not found in sister taxa [11]. A list of all of the PCR primers and probes are available in S1a and S1b Tables. The *fliC* and *fljB* flagella gene amplification primers from SGSA v. 1 were replaced with a common set of primers designed to co-amplify both genes using a common set of primers within the conserved 5’ and 3’ ends of the genes. Three multiplex PCR reactions were determined to be the minimum number of independent reactions to accommodate the 63 primer pairs used to amplify the targets of the assay and the compositions of the reactions are available in S1 Table.

The protocol for the SGSA is described previously with a few modifications [8]. The assay was modified to allow for template DNA in either the form of picked colonies or purified DNA. Colony based PCR was performed by selecting one colony from an overnight growth plate and using three separate 1 μL loops. 1/3 of the colony was added as template to each PCR reaction. Amplification conditions were as described previously [8], with the exception of the final elongation which was changed to 10 min at 72°C. Samples were hybridized to the SGSA array strips using a hybridization kit (Alere Technologies GmbH, Germany) and processed according to the manufacturer’s instructions, except the hybridization time was reduced to 30 min, and washing with C2 and C5 wash buffers was changed to 2 min, 30°C, 550 rpm.

### Limit of detection

Due to the potential of variability in DNA concentration arising from the DNA extraction kit procedure and due to potential differences attributable to colony size, we decided to examine the limits of the assay for both purified DNA template and cells. A single isolate of *Salmonella* ser. Typhimurium was selected to determine the limits of the assay since it is a biphasic serotype, which possesses many of the PCR targets that the SGSA is designed to detect. For the purified DNA template, the DNA was extracted using an EZ1 DNA Tissue kit (Qiagen) on an EZ1 Advanced automated robot (Qiagen). The DNA was quantified using the Qubit double stranded DNA broad range fluorometric quantification assay from Invitrogen. Seven independent serial dilutions of the *Salmonella* ser. Typhimurium template DNA at 10^−5^ of the starting template were used as input to the SGSA v. 2. The samples were then processed using the standard workflow and the serotype predictions were obtained from the array classifier. Colony detection limits were determined by selecting a single colony using a 1 μL loop and putting this into 1 mL of saline solution. This stock culture was gently mixed and was serially diluted to 10^−6^. Plate counts were obtained by plating 100 μL of each dilution onto a LB plate and counting the number of colonies present after an overnight incubation at 37°C. A sample of 2 μL of each dilution was used as input into the SGSA v. 2.

### Array classifier software

Previously, the probe hybridization patterns were analyzed using an Excel Macro to produce antigen and serotype predictions based on the highest signal intensity probes [8]. This approach possessed many limitations in distinguishing genetically similar flagellar antigenic complexes such as the “f/g-complexes” and e,n,x/en,x,z15/e,n,z15. A new approach to analyzing the probe signals was designed using a lazy learning k-nearest neighbor classification algorithm called the “array classifier” (https://github.com/jrober84/sgsa_array_classifier). The software is written in PHP and can be deployed as a web-based application. Based on a reference database of probe signals and antigens, the algorithm calculates the Euclidean distance between the query sample and all others in the database and selects the closest matches. A summed score for each antigen found within the top five matches was obtained by summing the inverse distance of each antigen and reporting the antigen with the lowest cumulative distance score. Each antigen is queried independently, and additional logic is included to accommodate antigens which are in both phases. In these cases, the flagellar probes are examined for both phase 1 and 2 to determine if any probes indicate an unambiguous placement of an antigen. For example, the “l-complex” can be in both phase 1 and 2 so it is possible that the initial predictions would find the best scoring antigen as an “l-complex” in both phases but biologically this does not occur. Therefore, the array classifier next subtracts the “l-complex” probes and then performs the classifications for both phases again thus refining the predictions. The results are compared, and should a compatible combination of alleles occur, then the “l-complex antigens” would be assigned to the corresponding phase compatible with the secondary antigen call. *S*. Brandenburg (B:l,v:e,n,z15) could initially be identified as B:l,v:lv but after subtracting the l,v probes the classification would be B:-:e,n,z15 so the l,v antigen is assigned to the first phase.

The newly adopted approach of the array classifier requires a database of patterns for the software to recognize. This initial database was created using a collection of draft genomes which had been downloaded from the SRA, assembled as described in Robertson et al. [12], and verified using the *Salmonella in silico* Typing Resource (SISTR). A collection of 1,311 genomes was selected to be the reference database so that all antigens with available genomic data had at least one representative in the database and the genomes were of high quality. High quality genomes are defined as the following: genome size between 4Mb to 6Mb, N50 > 100,000, intact and matching antigens detected by SISTR. Similar to the approach described in Braun et al. [7], the probe signals for each genome were simulated using blastn identities of the probe sequences. Their results demonstrated a high concordance between *in silico* and array hybridization and probe signals were converted into a binary presence and absence matrix [7]. In contrast to their approach, we divided the probe signals into four states (0, 0.3, 0.5, 0.8) based on the signal ranges previously seen for the SGSA [8]. A signal of zero was defined as any probe, which had more than 2 mismatches, or selective start base for the SSELLO probe was absent or a different base since the SSELLO procedure is designed to be SNP specific based on the 3’ base [8]. A perfect matching probe sequence would be assigned a score of 0.8 and one mismatched base would be scored as 0.5 with a score of 0.3 assigned to probes with 2 mismatches. blastn v. 2.2.31+ was performed on each genome using the probes as queries with a word size of 4. Using our array classifier script, each blast result was turned into a matrix representing the theoretical probe signal intensities. This training set was then interrogated using the larger set of verified genomes obtained from Robertson et al. [12] and the test genomes were classified into antigens and serotypes based on the reference database and the predictions were compared to what was reported on each genome.

### Wet-lab assessment of SGSA v. 2

A panel of 406 isolates representing 185 serotypes was selected to verify the performance of the SGSA and the isolate information is available in S2 Table. The samples were processed as described above and serotype predictions were obtained from the array classifier using the signal intensities produced by the IconoClust software on the Alere ArrayMate Reader (Alere Technologies). Biotin signal values greater than 0.7 for the experiment were considered valid. Positive signal values correspond to spot intensities above a minimum cut-off value of 0.25. The serotype predictions produced by the array classifier were compared to the traditional serotype assignments. Records were classified into six categories based on the results of the phenotypic serotype comparisons. If the predictions were fully concordant and the predicted serotype was unambiguous, it was assigned to **Type 0:** Full Match. If the antigens were fully concordant but the array was unable to produce a single serotype call, it was assigned to **Type 1:** Full antigen match, multiple serotypes. Should the antigenic calls be correct but an overall serotype call was incorrect, it was set to be **Type 2:** Full antigen match, incorrect serotype. **Type 3:** Matching partial antigen calls was assigned to records where part of the antigenic formula was correct, but antigens were missing. **Type 4:** Incorrect antigenic calls are the problematic category since it is the result of the array incorrectly identifying one or more antigens. If the array did not produce any antigenic calls the record was classified as **Type 5:** No antigenic calls.

## Results

### Array and workflow improvements

As described in Franklin et. al 2011(8), the SGSA v. 1 is able to detect 18 somatic serogroups: A (O:2); B (O:4); C1 (O:6,7); C2 (O:8); D (O:9); E (O:3); G (O:13); H (O:6,14); J (O:17); K (O:18); L (O:21); M (O:28); O (O:35); P (O:38); V (O:44); Y (O:48); O:58; and O:61. This comprehensive panel has been expanded to include: I (O:16), N (O:30), S (O:41) which completes the coverage of the most common serogroups in North America. Furthermore, the coverage of flagellar antigens was improved by the addition of 30 new probes to now cover 70% of flagellar antigens in the WKL. One of the shortcomings of the original array was ambiguity in the overall serotype prediction due to serotypes sharing the same antigenic formula based on serogroup. An additional 21 probes were added to the array to improve resolution of closely related serotypes.

**Fig 1:**
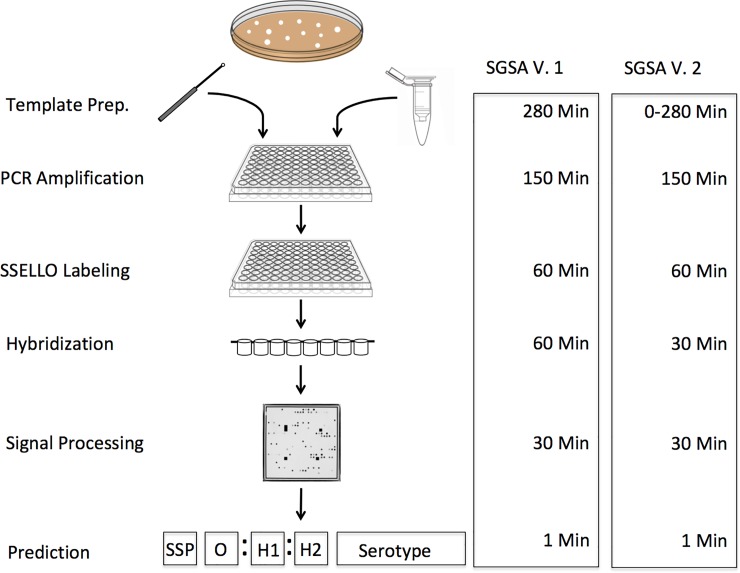
Workflow diagram outlining the process for both versions of the SGSA and the differences in processioning time between them. The workflow for the SGSA v. 2 consists of multiple small improvements, which increase the throughput of the assay and reduce the hands-on time required to process 96 samples. Colony PCR saves four hours of labor compared to DNA extraction on a 96 well plate, and up to 1.5 days considering time saved from not having to grow a broth culture from colonies.

Numerous process improvements have been made to the workflow of the SGSA v. 2 compared to its predecessor to reduce processing time and costs associated with running the assay. An overall process diagram comparing the two iterations of the SGSA is available in Fig 1. Parallel workflows using purified template DNA as well as PCR direct from picked colonies were introduced to provide flexibility to downstream users and to provide a more rapid and lower cost option for running the array. The use of picked colonies was found to be just as effective as purified template DNA or crude boiled lysates. The validation was performed using the colony-based method (data not shown). The hybridization times were also shortened to reduce the processing time without impacting the downstream results (data not shown).

### SGSA v. 2 limit of detection

**Fig 2:**
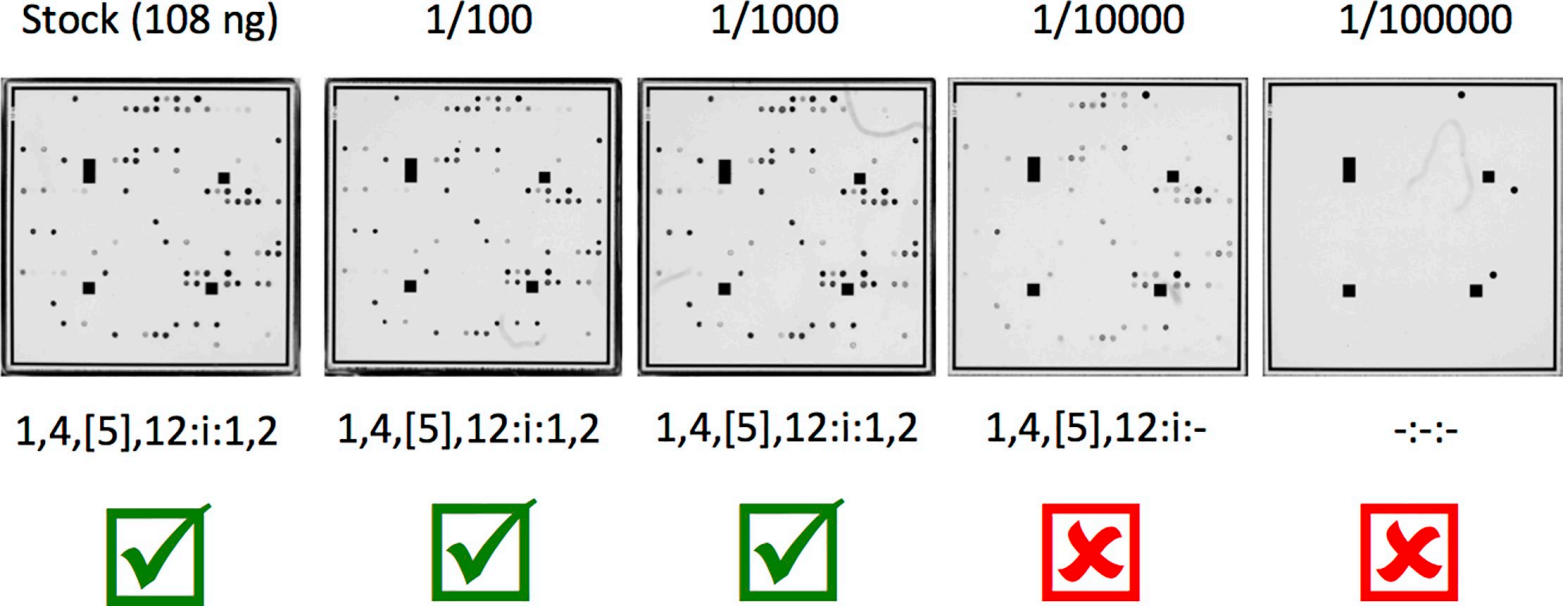
SGSA v. 2 array images processed by the Alere ArrayMate for the reference *Salmonella* ser. Typhimurium (1,4[5],12:i:-). A DNA stock was serially diluted and used as input into the SGSA v. 2 and imaged using the Alere ArrayMate system. The processed image files were used to create probe signal intensity files, which were used as input to the array classifier. Probe signals are consistent from the stock to a 10^−3^ dilution factor. Probe signals begin to be lost at 10^−4^ dilution and no signals are observed at 10^−5^ dilution. The limit of detection was set at 10^−3^ since it was the lowest dilution to completely identify the sample.

The SGSA v. 2 can be performed using both colonies and purified template and the limit of detection for the assay was assessed using both inputs. The array hybridization patterns were analyzed using the array classifier tool developed in this study to determine the point at which the platform failed to predict the serotype correctly. Based on the dilution series, the SGSA v. 2 provided a reliable identification with an input of 1.0 x 10^−1^ ng/μl with a standard deviation of 5.1 x 10^−2^. Probe signal intensities were consistent to a dilution factor of 1 x 10^3^ but susceptible to drop off rapidly on further dilution (Fig 2). Colony limit of detection was determined using seven independent serial dilution series for a single colony and colony counts were determined using plate enumeration and interpolated colony counts for plates, which were too numerous to count. The limit of detection for colonies was determined to be 860 CFU/mL with a standard deviation of 60. Based on the results from the DNA and colony limit of detection, the SGSA v. 2 is highly sensitive and can produce reliable identifications at low concentrations of cells and DNA template.

### *In silico* validation of SGSA v. 2 identifications

Previously validated *Salmonella* genome assemblies from Robertson et al. [12] with reported serotypes were used to test the serotype predictions of the SGSA v. 2. Blast identities of probes were converted into probe signals using the array classifier tool described in the Materials and Methods. Similar to the approach described in Braun et al. [7], a reference database was constructed using high quality genomes and their simulated probe signals, which covered the diversity of *Salmonella* serotypes and antigens represented in the public repositories. A total of 36,153 genomes were of sufficient quality to test the concordance of the SGSA v. 2 genoserotyping with phenotypic serotype assignment (S3 Table). The overall prediction results are summarized in Table 1 with fully concordant and unambiguous serotype assignment achieved for 31,924 (88.30%) of the tested samples. A further 3,916 (10.83%) of tested samples had fully concordant antigen predictions but multiple serotypes possessed the same formula, which could not be resolved further by the use of additional probes.

**Table 1:** Draft genome assemblies were queried for the presence of the SGSA v. 2 probe sequences and the sequence identities were converted into simulated probe signal values. These simulated values were processed using the array classifier and the antigenic formula and serotype were compared to the reported serotype for the assembly. Records were assigned to five different categories as described in the materials and methods.

Category	Number of Samples
Type 0: Full Match	31294
Type 1: Full antigen match, multiple serotypes	3916
Type 2: Full antigen match, incorrect serotype	11
Type 3: Matching partial antigen calls	524
Type 4: Incorrect antigenic calls	395
Type 5: No antigenic calls	13
**Total**	36153

The performance of the SGSA v. 2 classifications for serotypes with at least 100 representatives is summarized in Table 2. Complete results for the 368 serotypes tested *in silico* are available in the supplementary material (S4 Table). The highly prevalent serotypes of *Salmonella* (*i*.*e*. Enteritidis, Typhimurium, Heidelberg, Thompson, and I 1,4,[5],12:i:-) were all identified with 100% sensitivity and specificity except for *Salmonella* ser. Enteritidis and *Salmonella* ser. Typhimurium. A total of 7,841 *Salmonella* ser. Enteritidis samples were tested using the classifier and 97% of them were correctly identified. No false positive samples were found for *Salmonella* ser. Enteritidis. Of the 255 incorrectly typed *Salmonella* ser. Enteritidis isolates, 78% of the errors are the result of the classifier assigning a g,m,q H1 antigen instead of g,m which resulted in an identification of *Salmonella* ser. Blegdam (S3 Table). The three *Salmonella* ser. Typhimurium samples not correctly identified were partially identified due to missing either the 1,2 or i antigens.

**Table 2:** *In silico* performance of the SGSA v. 2 classifications based on simulated probe intensities for serotypes with at least 100 samples tested. Records were assigned to five different categories as described in the materials and methods.

*Salmonella* Serotype	Number of Samples	Sensitivity	Specificity	False Positives	Type 0: Full Match	Type 1: Full antigen match, multiple serotypes	Type 2: Full antigen match, incorrect serotype	Type 3: Matching partial antigen calls	Type 4: Incorrect antigenic calls	Type 5: No antigenic calls
Enteritidis	7841	0.97	1.00	3	7611	0	0	3	227	0
Typhimurium	6033	0.99	1.00	1	6029	0	0	4	0	0
Typhi	4344	1.00	1.00	0	4344	0	0	0	0	0
Kentucky	1459	1.00	1.00	0	1459	0	0	0	0	0
Newport	1265	1.00	1.00	0	0	1265	0	0	0	0
Heidelberg	1243	1.00	1.00	1	1243	0	0	0	0	0
Saintpaul	760	1.00	1.00	0	760	0	0	0	0	0
Infantis	741	1.00	1.00	0	738	0	0	3	0	0
I 1,4,[5],12:i:-	625	1.00	1.00	3	624	0	0	0	1	0
Paratyphi A	543	0.99	1.00	2	539	0	0	4	0	0
Weltevreden	536	0.97	1.00	0	522	0	0	14	0	0
Anatum	513	1.00	1.00	0	0	513	0	0	0	0
Agona	509	0.99	1.00	0	504	0	0	4	1	0
Hadar	487	1.00	1.00	0	0	487	0	0	0	0
Senftenberg	412	0.97	1.00	1	398	0	0	14	0	0
Paratyphi B	382	1.00	1.00	0	382	0	0	0	0	0
Montevideo	366	1.00	1.00	0	366	0	0	0	0	0
Braenderup	349	1.00	1.00	0	349	0	0	0	0	0
Dublin	329	0.99	1.00	1	326	0	0	0	3	0
Derby	306	1.00	1.00	1	306	0	0	0	0	0
Schwarzengrund	304	0.99	1.00	0	302	0	0	2	0	0
Mbandaka	288	1.00	1.00	0	287	0	0	0	1	0
Muenchen	288	1.00	1.00	0	0	288	0	0	0	0
Javiana	287	1.00	1.00	5	286	0	0	1	0	0
Virchow	260	1.00	1.00	0	260	0	0	0	0	0
Reading	226	1.00	1.00	0	226	0	0	0	0	0
Stanley	217	1.00	1.00	0	217	0	0	0	0	0
Oranienburg	209	0.98	1.00	5	204	0	0	5	0	0
Bareilly	190	1.00	1.00	0	190	0	0	0	0	0
Thompson	175	0.98	1.00	0	172	0	0	3	0	0
Cerro	163	0.99	1.00	0	0	162	0	1	0	0
Tennessee	151	1.00	1.00	0	151	0	0	0	0	0
Poona	124	0.00	1.00	0	0	0	0	1	123	0
Bovismorbificans	117	1.00	1.00	0	117	0	0	0	0	0
Mikawasima	109	0.99	1.00	0	108	0	0	1	0	0
Muenster	108	0.98	1.00	0	0	106	0	2	0	0
Give	103	1.00	1.00	0	0	103	0	0	0	0
Rissen	101	1.00	1.00	0	101	0	0	0	0	0
Albany	101	1.00	1.00	0	0	101	0	0	0	0

Out of the 368 serotypes tested *in silico* only 201 had at least three representatives. Using a threshold of 95% sensitivity, the array could reliably classify 198 serotypes with a specificity of at least 99% (S4 Table). With 92 false positives, Gallinarum (D:-:-) is a problematic serotype due to the inability of the classifier to distinguish between a failure to detect flagellar antigens and a *bona fide* Gallinarum (S4 Table). Of the 19 other serotypes tested with false positives, they all had 5 or fewer false positives. The SGSA v. 2 is more likely to incompletely type a sample rather than assign an incorrect serotype as evidenced by only 406 (1.12%) samples producing an incorrect identification and assigned to categories Type 2 and Type 4 (Table 1).

### SGSA v. 2 Wet-lab validation

Due to the high performance of the newly developed classifier and probe sequences *in silico*, a new version of the array was printed and validated using the improved colony PCR workflow. A panel of 406 samples representing 185 different serotypes was assembled to assess the performance of the newly developed array. Serotypes were included which possessed antigens that the array was not designed to detect in order to determine the analytical capacity of the array. The tested serotypes comprised 42 different O factors and 79 flagellar antigens (S2 Table). Predictions based on the SGSA v. 2 were compared to the reported serotype and antigens (S2 Table). Overall the SGSA v. 2 was able to correctly identify 60.89% of the panel completely with a further 18.31% correctly identified but with an antigenic formula matching multiple serotypes and the array was unable to refine them further (Table 3). For example, Newport (6,8,20:e,h:1,2) and Bardo (8:e,h:1,2) possess the same serogroup C2-C3 and antigens but are distinguished by the presence of specific O factors. Unfortunately, none of the probes on the array were able to separate these two serotypes. When the serotyping results of the *in vitro* and *in silico* experiments are compared, there are 112 serotypes that are unambiguously and reliably identified by the SGSA v. 2 with a further 45 where the antigens are reliably identified but additional testing is required to resolve the serotype completely.

**Table 3:** A panel of 406 isolates were analyzed using the SGSA v. 2 to determine the performance of the wet-lab assay. Serotype predictions were compared to the phenotypically reported serotype and grouped into one of six categories.

Category	Number of Samples
Type 0: Full Match	246
Type 1: Full antigen match, multiple serotypes	74
Type 2: Full antigen match, incorrect serotype	0
Type 3: Matching partial antigen calls	56
Type 4: Incorrect antigenic calls	27
Type 5: No antigenic calls	3
**Total**	406

## Discussion

*Salmonella* classification into serotypes is the essential first step for public health investigations and while many countries are poised to adopt WGS as a replacement to phenotypic serotyping, there are numerous groups where WGS will not be adopted in the near future. The costs and processing time for WGS have fallen dramatically but the $100–300 CAD price per sample and upwards of nine days to process a sample do not meet the needs of users which require low cost and rapid turnaround time. Previously, the cost of running the SGSA ($30) was compared to three other routinely used molecular methods for determining serotype: Salm SeroGen ($41), Check&Trace($42), xMAP($24) and was found to be the second least expensive molecular method [10]. Molecular methods such as the SGSA v. 2 can serve as a bridge technology for groups which want to take advantage of quicker processing times without the need for maintenance of numerous expensive antisera. The SGSA v. 2 is a mature technology, which has been ISO 17025 accredited and is being used routinely for the characterization of *Salmonella* within the Canadian OIE *Salmonella* Reference Laboratory at the National Microbiology Laboratory (Guelph, ON).

Building on previous work undertaken at our laboratory [8,13], the SGSA has undergone numerous improvements to expand the detection capabilities of the array and improve the workflow to reduce processing time and costs. Improvements to the laboratory workflow included a validation of a colony PCR that removes four hours of processing time and upwards of a complete day of technician time. Incremental improvements to the protocol also included minimizing wash and hybridization times to allow a single technician to complete 96 samples within a single working day (Fig 1). Additional O factor probes were designed to detect I (O:16), N (O:30), and S (O:41), and with the existing probes, the array is designed to detect the majority of serogroups. The complement of flagellar probes was also increased with 30 additional probes, which now cover more than 70% of the described flagellar antigens. Due to variability in colony size and DNA concentration we examined the ability of the SGSA v. 2 to detect highly diluted samples. We found that the array is highly sensitive to low inputs of cells or DNA template (Fig 2). Due to the high sensitivity of the array, the array will produce reliable results even on highly dilute samples and variability in starting material should not pose an issue for the platform.

The performance of the SGSA v. 2 was improved by the development of a new analysis tool termed the “array classifier”. This software employs a weighted k-nearest neighbor classification algorithm to determine the closest matching antigen with some additional logic to utilize serotype specific probes to refine serotype predictions. This approach examines each antigen separately as opposed to the Pattern Match algorithm described previously in Braun et al. [7], which uses the complete set of probes to match to a serotype to identify the closest matching serotype. A reference database was constructed using 1311 draft *Salmonella* genomes whose serotype designation was confirmed *in silico* using SISTR [14]. With the unprecedented amount of genomic data available for *Salmonella*, we were able to validate the serotype predictions of the SGSA v. 2 with a dataset of unrivaled sized for molecular serotyping. The pattern match DNA serotyping assay described in Braun et al. [7] used 168 genome sequences to benchmark the performance of their algorithm. In the current study, a total of 36,153 genome assemblies met the quality to test the concordance of the SGSA v. 2 molecular serotyping with phenotypic serotype assignment (S3 Table).

The dataset was highly biased towards six serotypes: Enteritidis, Typhimurium, Typhi, Kentucky, Newport, and Heidelberg due to their relevance in public health; which account for 61% of the samples tested (Table 2). Out of 7841 Enteritidis genomes, we obtained a correct identification 97% of the time with no instances of samples incorrectly called Enteritidis (Table 2). Typhimurium was the second most abundant serotype with 6033 genomes and similar to Enteritidis, the SGSA v. 2 produced concordant predictions for 99% of the genomes tested (Table 2). For the remaining four top serotypes in our dataset, the SGSA v. 2 produced concordant predictions for 100% of them with 100% specificity. The SRA dataset contained 368 different reported serotypes but only 201 of these contained at least three representatives. We found that at a threshold of 95% sensitivity and 99% specificity, the SGSA v. 2 was able to reliably identify 198 of the 201 serotypes *in silico*.

The power of the SGSA v. 2 is the antigen-based detection approach, which theoretically allows for the detection many more serotypes based on the identification of the individual antigens. Using the same thresholds of 95% sensitivity and 99% specificity with three samples per antigen, the SGSA v. 2 is able to reliably identify 16 different serogroups and 35 flagellar antigens (S3 Table). Based on the *in silico* results, the SGSA v. 2 can theoretically identify 988 of the 2709 serotypes described in the WKL [4] with 198 serotypes confirmed to work with the SGSA.

The highly promising *in silico* validation of the SGSA v. 2 were verified *in vitro* using a panel of 406 isolates representing 185 serotypes possessing a wide diversity of antigens. The SGSA v. 2 was able to confirm the results of the *in silico* experiments demonstrating nearly identical performance on the serotypes tested. The panel was designed to stress test the array to ensure that the array would perform well even on samples possessing antigens the array was not designed to detect. The array was able to unambiguously identify 60.89% of the samples with a further 18.31% of samples identified with a correct antigenic formula but multiple serotypes. Depending on the needs of the laboratory, additional testing may be required to identify serotype variants. Taken with the extensive *in silico* validation of the SGSA v. 2, the platform is able to serve as a rapid diagnostic tool for serotyping both common and rare *Salmonella* serotypes. The OIE *Salmonella* Reference laboratory, is effectively utilizing the SGSA v. 2 in combination with conventional serotyping as a high throughput alternative for serotype determination for time sensitive and high-volume projects.

### Conclusions

The SGSA v. 2 is a significant improvement over the first version and is confirmed to identify 112 different serotypes with the potential to identify up to 988 based on the 16 serogroups and 35 flagellar antigens the array has been verified to identify *in silico*. The array-based platform of the SGSA is a mature, cost-effective and ISO accredited test which is a suitable replacement for phenotypic serotyping for laboratories not ready or able to adopt WGS.

## Supporting information

S1 Table Primers and probe sequences for the SGSA v. 2. (XLSX)

S2 Table Reported and predicted serotypes for isolates used for testing the SGSA v. 2 in vitro protocol. (XLSX)

S3 Table Reported and predicted serotypes for publicly available NCBI Illumina WGS isolates used for testing the SGSA v. 2 in silico protocol. (XLSX)

S4 Table *In silico* performance summary of the SGSA v. 2 on Illumina based on serotype. (XLSX)
